# Transcriptomic responses in the blood and sputum of cigarette smokers compared to e-cigarette vapers

**DOI:** 10.1186/s12931-023-02438-x

**Published:** 2023-05-18

**Authors:** Mario F. Perez, Marina Yurieva, Spandana Poddutoori, Eric M. Mortensen, Laura E. Crotty Alexander, Adam Williams

**Affiliations:** 1 Department of Pulmonary, Critical Care and Sleep Medicine, University of Connecticut School of Medicine, Farmington, CT USA; 2 The Jackson Laboratory for Genomic Medicine, Farmington, CT USA; 3 AXA Life and Health Re US, Inc., Boston, MA USA; 4 Division of Pulmonary Critical Care, Department of Medicine, University of California San Diego, San Diego, CA USA; 5 Division of Allergy and Immunology, Department of Medicine, Northwestern University Feinberg School of Medicine, Chicago, IL USA

**Keywords:** Transcriptomics, Tobacco, Immune cells of the airways, Electronic cigarettes, Cigarette smoking

## Abstract

**Rationale:**

Electronic (e)-cigarettes are popular among youth and cigarette smokers attempting to quit. Studies to date have focused on the utility of e-cigarettes as a smoking cessation tool, but the biological effects are largely unknown.

**Objectives:**

To identify transcriptomic differences in the blood and sputum of e-cigarette users compared to conventional cigarettes smokers and healthy controls and describe biological pathways affected by these tobacco products.

**Methods:**

Cross-sectional analysis of whole blood and sputum RNA-sequencing data from 8 smokers, 9 e-cigarette users (e-cigs) and 4 controls. Weighted gene co-network analysis (WGCNA) identified gene module associations. Ingenuity Pathway Analysis (IPA) identified canonical pathways associated with tobacco products.

**Main results:**

In blood, a three-group comparison showed 16 differentially expressed genes (DEGs); pair-wise comparison showed 7 DEGs between e-cigs and controls, 35 DEGs between smokers and controls, and 13 DEGs between smokers and e-cigs. In sputum, 438 DEGs were in the three-group comparison. In pair-wise comparisons, there were 2 DEGs between e-cigs and controls, 270 DEGs between smokers and controls, and 468 DEGs between smokers and e-cigs. Only 2 genes in the smokers vs. control comparison overlapped between blood and sputum. Most gene modules identified through WGCNA associated with tobacco product exposures also were associated with cotinine and exhaled CO levels. IPA showed more canonical pathways altered by conventional cigarette smoking than by e-cigarette use.

**Conclusion:**

Cigarette smoking and e-cigarette use led to transcriptomic changes in both blood and sputum. However, conventional cigarettes induced much stronger transcriptomic responses in both compartments.

**Supplementary Information:**

The online version contains supplementary material available at 10.1186/s12931-023-02438-x.

## Introduction

The use of e-cigarettes or “vaping” is popular among adolescents, young adults as well as former and current cigarette smokers [1]. Although e-cigarettes with nicotine are a potential tool for smoking cessation, their effectiveness remains controversial [2], [3]. The benefit of switching from conventional tobacco to e-cigarettes relies on the notion that they are less harmful than combustible cigarettes. Whereas smoke from cigarettes contains 4,000–7,000 chemicals, with many known harmful toxicants and carcinogens, e-cigarette aerosols contain many fewer (50–120) chemicals [4]. Nevertheless, epidemiological studies have linked e-cigarette use with pulmonary diseases known to occur with conventional cigarette smoking, namely bronchitis, COPD, hypersensitivity pneumonitis, eosinophilic pneumonia, lipoid pneumonia, and asthma [5–7], and in 2019, the Center for Disease Control and Prevention (CDC) identified a novel disease caused by vaping: e-cigarette or vaping product use-associated lung injury (EVALI) [8]. Beyond our clinical experience, little is known about the effects of e-cigarette use on the respiratory system, particularly when compared to conventional tobacco.

There are several reports from both in vitro and human subject studies showing that exposure to e-cigarette aerosols (commonly referred to as “vapor”) leads to significant changes in gene expression by airway epithelial cells [9–11]. However, to our knowledge, there are no reports regarding the effects of e-cigarette use on gene expression in immune cells in human airways. There are scarce reports of gene expression in sputum of healthy smokers, and the most robust, which was funded by the tobacco industry, showed that cigarette smoking alters two biological pathways: (1) the xenobiotic response and oxidative stress; and (2) immune related responses [12]. More recently, a study of inflammatory markers in the sputum of e-cigarette users showed that newer generation e-cigarettes (4th generation) may cause more immunosuppression than prior e-cigarette devices; nevertheless, the effects of e-cigarettes on human airway and systemic immunity remain unclear.

The goals of this work were to identify biological pathways differentially regulated in the blood and sputum of e-cigarette users when compared to cigarette smokers (CS) and healthy controls and to shed light on how exposure to these tobacco products may lead to respiratory diseases. Our primary hypothesis was that these two disparate inhalants would lead to gene expression changes in the sputum and the circulation reflecting homeostatic changes in multiple biological pathways contrasting with those induced by conventional tobacco.

## Methods

### Human subjects

**Fig. 1:**
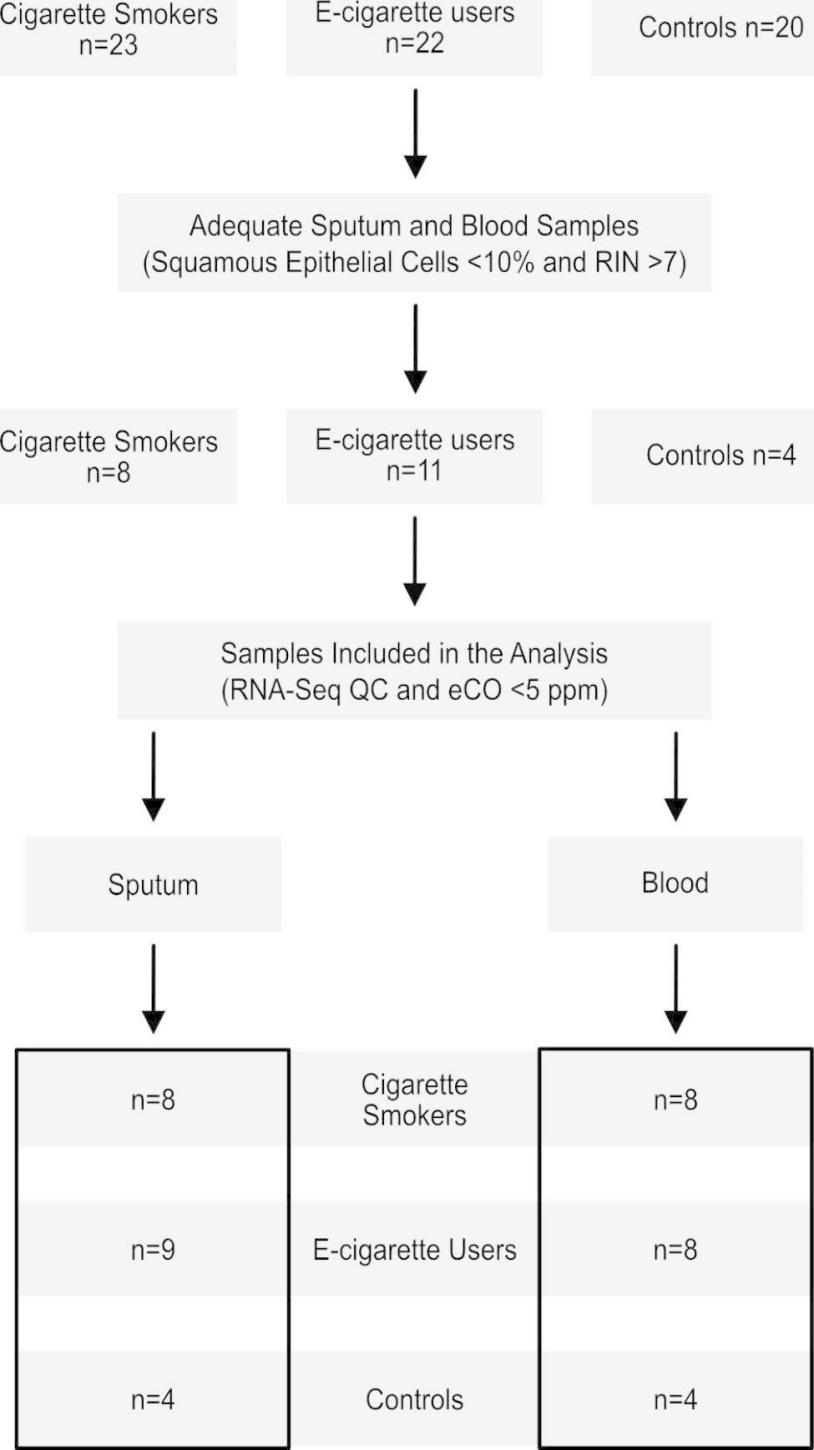
Diagrammatic representation of the study cohort and workflow. A total of 65 participants were enrolled in the study; all underwent exhaled carbon monoxide (eCO) measurement, spirometry, sputum induction with hypertonic saline, and blood draw. Adequate (i.e., with less than 10% squamous cells) sputum samples were obtained from 33 subjects. Good quality RNA (RNA integrity numbers (RIN) > 7) was obtained from 23 samples, which then were matched with whole blood RNAs from the same subjects to comprise our final study cohort. Following RNA-Seq and principal component analysis, additional samples were excluded (see main text and Fig. 2) before in-depth analysis of differentially expressed genes. Only smokers and controls with eCO of < 5 ppm were included in the final analysis

Sixty-five participants were enrolled between May 1, 2017, and March 1, 2020 (Fig. 1). Inclusion criteria included age 18–55 years old plus exclusive daily use of e-cigarettes or conventional cigarettes for at least one year. Exclusion criteria included use of prescription medications (excluding birth control pills); previously diagnosed pulmonary disease; emergency room visits or hospitalization within the prior year; pregnancy; history of allergic rhinitis/rhinosinusitis, chemical exposure (including dust and wood) or an adverse reaction to albuterol; and current use of cigars, pipes, hookah, chewing tobacco or other tobacco products, marijuana, cocaine, or illicit drugs. We used these stringent criteria to avoid misclassification and residual bias, as older subjects may have greater significant undiagnosed comorbidities, while other noxious exposures, such as viral infections or allergies, could affect the biological pathways similarly to the use of tobacco products. All subjects underwent informed consent. This study was approved by the University of Connecticut Institutional Review Board.

### Sputum induction, blood collection and RNA extraction and processing

All 65 participants underwent exhaled carbon monoxide (eCO) measurement, spirometry and sputum induction with hypertonic saline [13]. In brief, subjects were asked to blow their nose and to complete three 7-minute cycles of inhalation of 3%, 4%, and 5% hypertonic sodium chloride delivered by an ultrasonic nebulizer (DeVilbiss®). At the end of each cycle subjects were encouraged to expectorate into a sterile cup; samples were processed within 15 min of collection. Sputum plugs were selected, and then cellular and aqueous compartments were separated using dithiothreitol (DTT) and centrifugation as previously described [13], [14]. Cell viability was determined by trypan blue exclusion. Total cell counts were determined by hemocytometer, and final differentials were performed on Wright-Giemsa stained Cytospin® slides of ≥200 cells 15–18. Sputum samples with greater than 10% squamous cells were excluded as higher squamous cells concentration may reflect salivary contamination and may impact biomarker measurements [19], [20]. Aqueous phase was aliquoted and stored at -80 °C; cell pellets were stored at -80 °C in RNAlater®. YKL-40, TNF-alpha, interleukin (IL)-6, IL-33, IL-8, IL-10, and IL-13 were measured using the commercially available Magnetic Luminex Assay (R&D Systems Inc., Minneapolis, MN) and Bio-Plex 200 reader (BioRad Laboratories, Hercules, CA). All samples were tested in duplicate as described elsewhere [21]. RNA extraction was performed using the miRNAeasy and RNeasy MinElute kits (Qiagen) according to manufacturer instructions. Phlebotomy was performed and blood underwent automated cell blood count by the clinical laboratory, and RNA extraction and analysis via PAXgene® RNA tubes (BD Biosciences) and PAXgene blood RNA kit (Qiagen).

### RNA-Sequencing and qRT-PCR experiments

RNA from sputum and blood was analyzed on an Agilent TapeStation 4200 (Agilent Technologies) using the RNA High Sensitivity assay. Only samples with ribosomal integrity numbers (RIN) values above 7.0 were considered for library preparation. Samples from blood and sputum were processed as one batch respectively. RNA samples were prepared for mRNA-Sequencing by the University of Connecticut Genomic Core Facility (Institute for System Genomics) using the Illumina TruSeq Stranded mRNA Sample Preparation kit, following the manufacturer’s protocol. Samples were combined into one sequencing pool and run as one sample on an Illumina HiSeq 2500 as paired end 100 bp reads. 100 ng of sputum RNA was reversed transcribed to synthesize cDNA to then perform real time PCR using Applied BioSystems 7900HT system as directed by manufacturer. Commercially available primer/probes were used for validation (see Supplemental Table 1). Relative levels of each gene were normalized against four control genes (*HPRT*, *18s*, *ACTIN* and *GAPDH*). All sequencing data presented in this publication have been deposited in NCBI’s Gene Expression Omnibus and are accessible through GEO Series accession number GSE223736.

### Statistical analysis and bioinformatics approach

Participant characteristics were analyzed using chi-square for categorical variables and non-parametric methods for continuous variables. A two-tail p < 0.05 was considered significant. For differential gene expression an adjusted (Benjamini and Hochberg [BH] method) p-value of ≤ 0.05 was considered significant. Quality control of the fastq files was performed using FASTQC [22]. BBDuk [23] was used to remove hemoglobin contamination from the blood samples. The reads were mapped to gencode.v38 release (GRCh38.p13 Release 38) using Salmon [24]. R version 4.1.2 was used for the statistical analysis [25]. For the analysis of the qRT-PCR we log-transformed and plotted the data as a heatmap using R software pheatmap package (Version 1.0.12). R packages DESeq2 (1.32.0), tximport (1.20.0), Salmon (version 1.5.2) and WGCNA (1.4.1717) 26–29 were used for quantification and analysis of the RNA-sequencing data. A cut-off of 95% quantile was used to subset the genes for Weighted Gene Co-expression Network Analysis (WGCNA). WGCNA was performed in a signed network and soft thresholds to the similarity matrix with the best power chosen to simulate a scale free network. Three WGCNA were performed using the differentially gene expression of the following three comparisons: (1) E-cigarettes vs. controls, (2) Cigarette vs. controls and (3) Cigarette vs. E-cigarettes. The gene clusters/modules from the WGCNA were further related with the metadata and phenotypical characteristics of interest, including eCO level, cotinine exposure, pulmonary function tests and cell counts in both blood and sputum. Canonical pathways were generated through the use of Ingenuity Pathway Analysis® (IPA) (Qiagen) [30].

## Results

### Population and biological samples

#### Study participants

A total of 65 participant were enrolled in our study [18]. Details and demographic characteristics of this entire cohort have been reported elsewhere [13]. Adequate (i.e., less than 10% squamous cells) sputum samples were obtained from 33 subjects. Good quality RNA (RIN ≥ 7) was obtained from 23 samples, which were matched with whole blood RNAs (also RIN ≥ 7) from the same subjects to comprise our final study cohort (Fig. 1). The demographic characteristics of these 23 subjects are presented in Table 1. Nine subjects used e-cigarettes exclusively daily with a median of 2 years, while 8 subjects were exclusive daily conventional smokers with a median of 13 pack-years. There were no significant differences among groups with respect to age, marital status, race, oxygen saturation, or systolic blood pressure. We did not find any significant differences in the selected inflammatory markers (cytokines) measured in the population in both sputum and serum (Supplemental Table 2). As expected, cigarettes smokers (CS) had significantly higher eCO levels when compared to e-cigarette users and controls (Median 11.0 vs. 1.1 and 1.5 ppm respectively [p = 0.004]). All subjects had normal and comparable airflow as measured by spirometry. Serum cotinine and nicotine dependence, assessed using the PENN (E) Cigarette Dependence Index, were similar for e-cigarette users and CS (Median 95.3 vs. 68.2 and 5.0 [p = 0.271] and 15.5 vs. 15.0 [p = 0.532] respectively). Among E-cigarette users, six used 4th generation e-cigarettes, including JUUL®, and all reported using “e-juice” containing nicotine concentrations greater than 10 mg/ml. Menthol and fruity flavors were the most common flavors reported, with four subjects using each (Supplemental Table 3).

**Table 1:** Characteristics of the study participants with matched RNA-seq data from sputum

	Cigarette Smokers (8)	E-cigarette Users (11)	Controls (4)	p-value
Age (years)	32.50 [26.50, 43.25]	24.00 [21.50, 28.50]	29.00 [25.75, 37.00]	0.125
Female/Male	2/6 (25.0/75.0)	2/9 (18.2/81.8)	1/3 (25.0/75.0)	1
White Race	7 (87.5)	11 (100.0)	3 (75.0)	0.15
Never Married	5 (62.5)	8 ( 72.7)	3 (75.0)	1
Body Mass Index	25.47 [22.52, 31.75]	24.71 [23.68, 34.37]	24.22 [21.02, 28.36]	0.606
Heart Rate	77.00 (16.91)	73.00 (7.92)	71.25 (8.30)	0.676
Systolic Blood Pressure*	118.75 (9.19)	127.82 (10.84)	120.25 (19.33)	0.252
Diastolic Blood Pressure*	74.62 (6.25)	78.00 (8.65)	75.00 (11.22)	0.653
Oxygen Saturation (%)	97.88 (1.25)	98.73 (1.49)	99.50 (0.58)	0.134
Carbon Monoxide (ppm)	11.00 [7.75, 15.75]	1.10 [1.00, 2.50]	1.50 [1.00, 2.00]	0.004
PENN Score**	15.50 [13.50, 18.00]	15.00 [13.50, 16.00]	-	0.532
Serum Cotinine (ng/ml)	95.25 [89.95, 97.45]	68.15 [20.08, 91.78]	5.00 [5.00, 43.65]	0.09
FEV1 (% Predicted)	91.50 [89.25, 95.00]	101.00 [87.50, 106.50]	96.00 [87.50, 104.75]	0.577
FVC (% Predicted)	91.50 [86.00, 100.25]	99.00 [88.50, 111.00]	93.50 [86.25, 100.50]	0.481
FEV1/FVC Ratio	78.50 [71.75, 84.50]	81.00 [78.50, 87.00]	83.50 [80.50, 87.50]	0.573

Continues variables presented as median and interquartile range [IQR] and Categorical variables presented as count and percentage (%)

* mmHg **Penn State (e) cigarette dependence index not assessed in controls, p-value represents Wilcoxon rank-sum test

#### Blood sample characteristics

Controls had a lower proportion of circulating neutrophils when compared to CS (60.9 vs. 48.3% [p = 0.028]) and e-cigarette users (60.9 vs. 55.9% [p = 0.043]) but a three-group comparison was not significant (Table 2). There were no other significant differences among other circulating blood cells, total white blood cells (WBCs), platelet count or hemoglobin concentration.

**Table 2:** Blood and Sputum cell counts with differential cell populations

	Cigarette Smokers (8)	E-cigarette Users (11)	Controls (4)	p-value
**Blood Measurements**				
Hemoglobin (mg/dl)	14.25 [13.78, 15.53]	15.20 [13.85, 15.95]	14.35 [13.65, 15.13]	0.846
Hematocrit (%)	42.2 [40.88, 44.75]	44.9 [41.65, 47.25]	42.35 [31.31, 44.85]	0.651
Platelet Count * 10^3/mcL	251.0 [221.00, 273.75]	263.0 [240.50, 307.50]	209.5 [202.25, 240.00]	0.509
White Cell Count * 10^3/mcL	7.9 [6.20, 8.88]	5.9 [4.45, 7.25]	4.5 [4.00, 5.05]	0.118
Neutrophils (%)	60.90 [53.25, 67.38]	55.90 [53.50, 62.10]	48.25 [44.27, 50.88]	0.061
Lymphocytes (%)	27.35 [21.03, 34.83]	29.40 [27.75, 31.25]	38.1 [33.38, 43.23]	0.083
Monocytes (%)	7.7 [5.12, 8.47]	9.9 [7.70, 10.55]	9.55 [7.27, 12.05]	0.096
Eosinophils (%)	2.85 [2.05, 3.70]	1.9 [1.40, 3.10]	3.4 [2.88, 4.18]	0.352
Basophils (%)	0.55 [0.48, 0.60]	0.7 [0.55, 0.85]	0.7 [0.67, 0.97]	0.088
**Sputum Measurements**				
Sputum Plug Weight (mg)	125.0 [67.50, 175.00]	130.0 [60.00, 240.00]	80.0 [30.02, 120.00]	0.342
Cell Viability (%)	95.0 [92.25, 97.25]	96.4 [95.00, 97.75]	96.0 [95.75, 96.92]	0.459
Total Cells *10^6/g	4.92 [4.48, 6.87]	6.46 [3.02, 23.72]	6.83 [4.46, 14.16]	0.81
Neutrophils (%)	22.75 [17.25, 38.65]	25.0 [15.00, 41.00]	26.2 [21.05, 32.92]	0.876
Macrophages (%)	71.0 [58.50, 78.25]	68.3 [49.50, 79.00]	67.35 [62.90, 72.78]	0.949
Lymphocytes (%)	0.0 [0.00, 0.15]	0.25 [0.00, 1.00]	0.0 [0.00, 0.40]	0.572
Eosinophils (%)	0.7 [0.30, 1.00]	0.25 [0.00, 1.00]	0.0 [0.00, 1.00]	0.538
Epithelial cells (%)	1.20 [0.48, 2.60]	1.00 [0.25, 2.85]	0.0 [0.00, 0.00]	0.108

% Percent of the cell populations presented as median and interquartile range [IQR]

#### Characteristics of Induced Sputum

Although the amount of sputum produced and processed from healthy controls tended to be lower (Median 80.0 mg) than that from CS (125 mg) and e-cigarette users (130 mg), this was not statistically significant (Table 2). Similarly, there were no differences in total number of cells, viability, or proportion of neutrophils, macrophages, lymphocytes, eosinophils, and bronchial epithelial cells which is consistent with what other studies have reported [31], [32].

#### RNA-Seq Quality Control

**Fig. 2:**
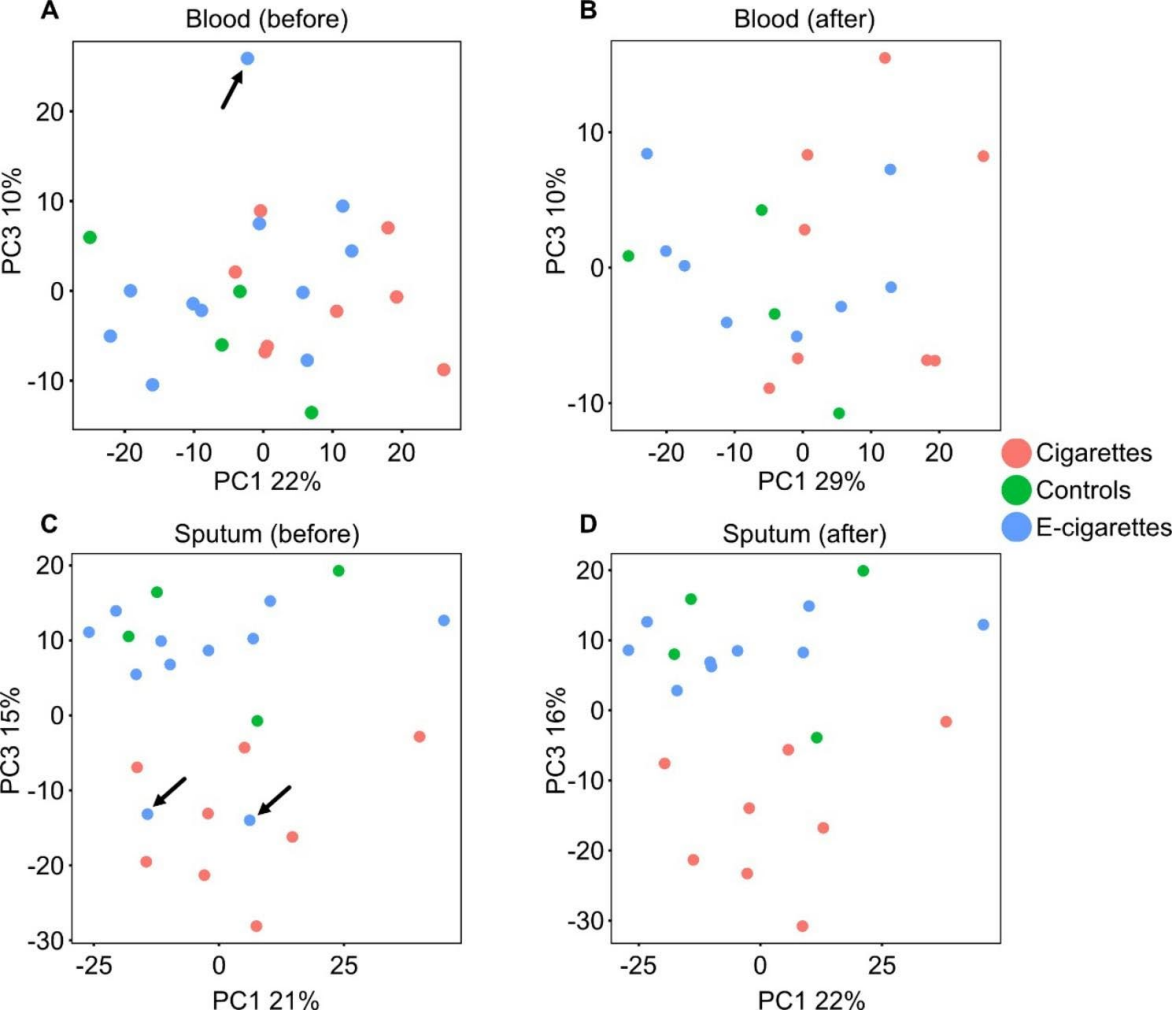
Principal component analysis reveals outliers and dual users. Principal component analysis (PCA) of RNA-Seq data from all (A) blood samples and (C) sputum samples. (A) A single e-cigarette blood sample was deemed as an outlier (black arrow) and excluded from further blood analysis (note this sample was retained in the sputum group). (C) Two sputum samples from e-cigarette users (black arrows) were observed to cluster with samples from smokers. Analysis of exhaled carbon monoxide in these subjects reveled levels of more than 5 ppm, suggesting that they likely were dual e-cigarette and tobacco users. Samples from these subjects were excluded from further analysis for both blood and sputum. PCA plots for (B) blood and (D) sputum following exclusion of outliers and dual users

Quality control of the RNA-Seq data was acceptable for all whole blood (hereinafter referred to as blood) samples except from one e-cigarette user, which was removed from further analysis (Fig. 2A). RNA-Seq quality controls showed that all sputum samples were acceptable for further analysis. Surprisingly, principal component analysis showed that two e-cigarette user samples clustered with CS. Although these subjects identified as exclusive e-cigarette users, their eCO levels were greater than 4 ppm (9 and 19 ppm), suggesting in retrospect that they are dual users. Thus, these subject samples were also removed from further transcriptomic analysis (Fig. 2B).

### Transcriptomic differences in blood

#### Differentially expressed genes in blood

A three-group comparison (Likelihood Ratio Test [LRT]) showed 16 differentially expressed genes (DEGs) (Supplemental Table 4). Eleven genes (*RMRP, LCN8, RBPMS2, WASHC1, ZNF703, STON2, IGHV7-4-1, ICAM4, CHRNA2, SAMD14, ATF5*) were overexpressed and 5 genes (*WDR27, CBSL, RP11-807H22.10, SIX4, HERC2P2*) were underexpressed. No specific biological pathways were overrepresented with this set of genes.

#### DEGs in the blood of E-cigarette users vs. controls

**Fig. 3:**
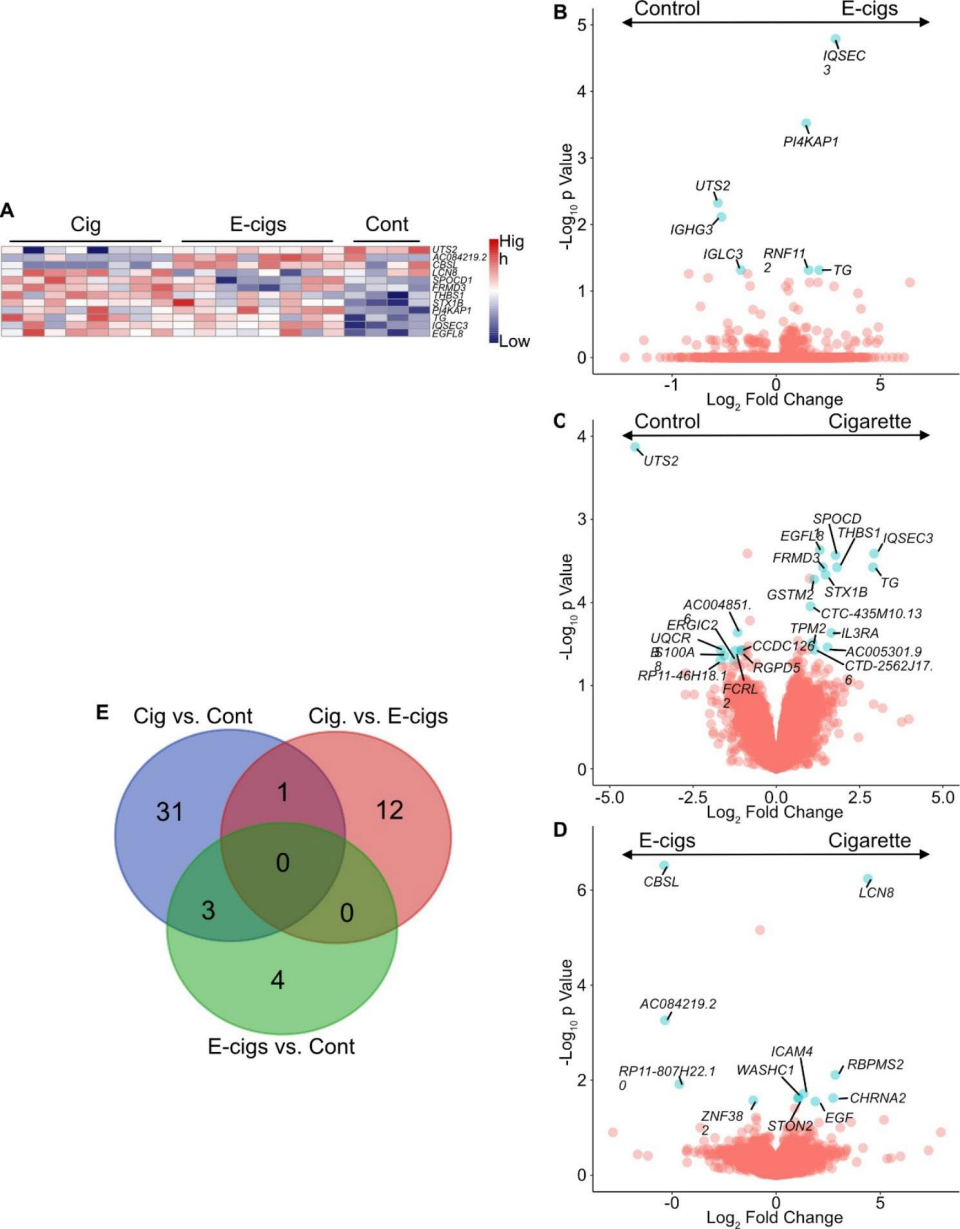
Increased numbers of differentially expressed genes in the blood of cigarettes smokers vs. e-cigarette users. (A) Heatmap showing significant (log2 fold change |≥1 and p≤ 0.005) combined genes from pairwise comparisons in blood (cigarette smokers, Cig; e-cigarette users, E-cigs; and controls, Cont). (B, C and E) Volcano plots of DEGs in blood from pairwise comparisons between (B) e-cigarette users (E-cigs) and controls, (C) cigarette smokers and controls, and (E) cigarette smokers and E-cigarette users (E-cigs). (D) Venn diagram representing the number of DEGs that overlap between each comparison

Comparison of e-cigarette users and controls revealed 7 DEGs in blood (Fig. 3A-B, Supplemental Table 4). Three genes (*UTS2*, *IGHG3*, and *IGLC3*) were underexpressed, while 4 (*PI4KAP1*, *RNF112*, *TG*, and *IQSEC3*) were overexpressed in e-cigarette users. Similar to the above comparison there were no specific biological pathways overrepresented with these genes.

#### DEGS in the blood of cigarette smokers vs. controls

In contrast to the e-cigarettes vs. controls, CS had a more robust effect on blood gene expression with 35 DEGs (Supplement Table 4; Fig. 3A C). Three of these (*IQSEC3*, *TG* and *UTS2*) also were differentially expressed in the comparison between e-cigarette users and controls (Fig. 3D). Expression of these genes correlated with the use of tobacco products, with *IQSEC3* and *TG* overexpressed and *UTS2* underexpressed in blood of tobacco product users as compared to controls (Fig. 3B-C).

#### DEGS in the blood of cigarette smokers vs. E-cigarette users

Comparison of CS to e-cigarette users revealed 13 DEGs (see supplemental Tables 4, Fig. 3E). Eight genes were overexpressed (*LCN8, RBPMS2, ICAM4, CHRNA2, STON2, WASHC1, EGF, ZNF703*) in the blood of CS when compared to e-cigarette users. Zinc Finger Protein 703 (*ZNF703*) also was overexpressed in CS compared to controls (Fig. 3D). Although no biological pathways were enriched by these genes, in the blood CS had greater transcriptomic changes than e-cigarette users and controls.

### Transcriptomic differences in sputum

#### DEGs in the sputum

Comparison of all 3 groups (LRT) revealed a total of 438 differentially expressed genes (Supplemental Table 4). And similar to the DEG analysis of the blood, these differences were driven mostly by CS.

#### DEGs in the sputum of E-cigarette users vs. controls

**Fig. 4:**
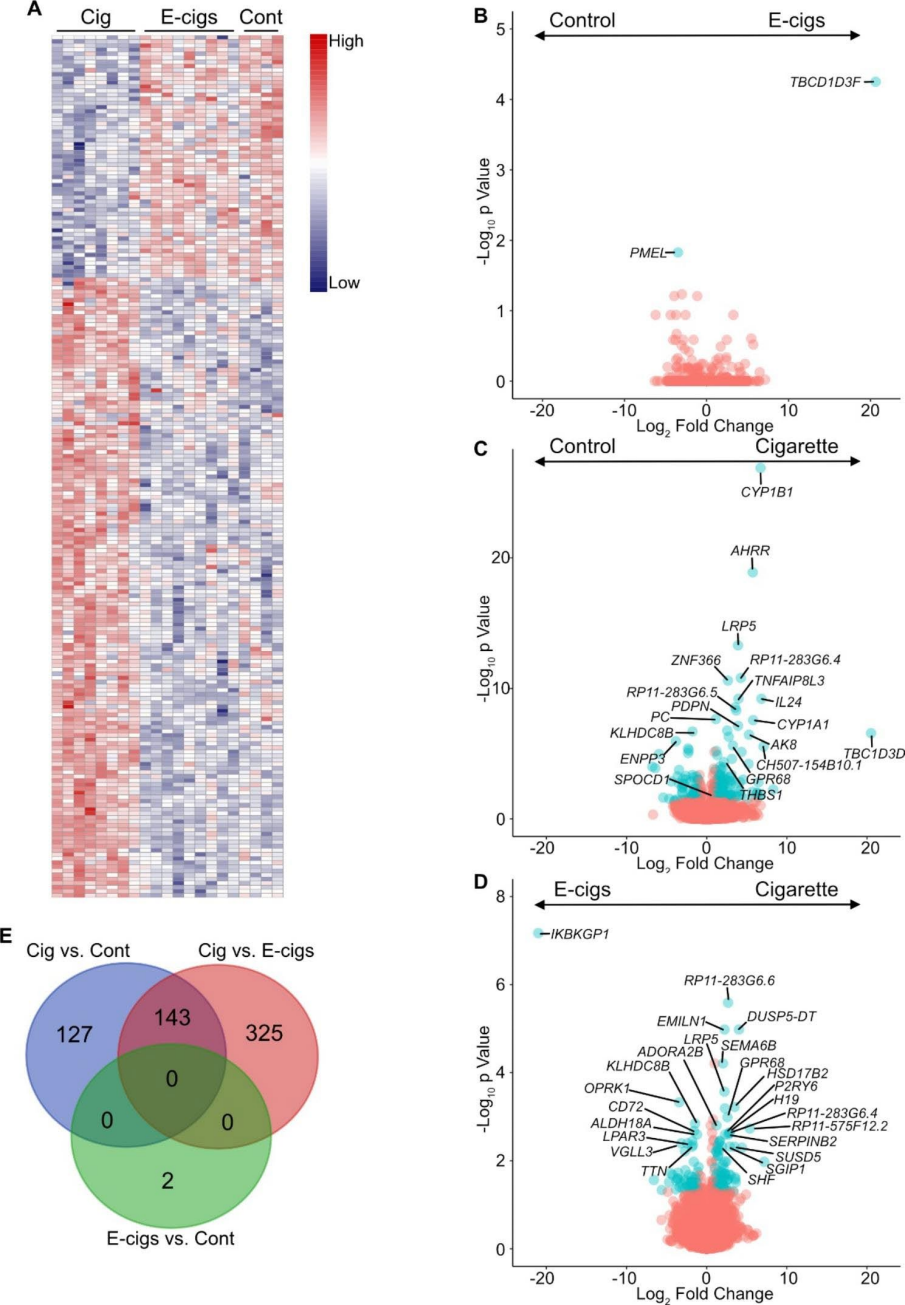
Increased numbers of differentially expressed genes in the sputum of cigarettes smokers vs. e-cigarette users. (A) Heatmap showing significant (log2 fold change |≥1 and p≤ 0.005) combined genes from pairwise comparisons in sputum (cigarette smokers, Cig; e-cigarette users, E-cigs; and controls, Cont). (B, C and E) Volcano plots of DEGs in sputum from pairwise comparisons between (B) e-cigarette users (E-cigs) and controls, (C) cigarette smokers and controls, and (E) cigarette smokers and E-cigarette users (E-cigs). (D) Venn diagram representing the number of DEGs that overlap between each comparison

Only 2 genes were differentially expressed (*PMEL* and *TBC1D3F*) between e-cigarette users and controls (Fig. 4A-B). *PMEL*, a gene involved in lymphangioleiomyomatosis (LAM) [33] was underexpressed. *TBC1D3F*, which is involved on macropinocytosis and tissue repair [34] was overexpressed in the sputum of e-cigarette users.

#### DEGs in the sputum of cigarette smokers vs. controls

A total of 270 genes were differentially expressed in the sputum between CS and controls (Fig. 4C and Supplemental Table 4). Interestingly, of these 270 genes, 2 (*THBS1* and *SPOCD1*, (Supplemental Fig. 1 and Supplemental Table 5) were overexpressed in both the blood and sputum of CS compared to controls. There was no overlap with genes differentially expressed between e-cigarette users and controls (Fig. 4D). Overexpressed genes in this set were overrepresented in biological pathways that encompass processes from oxidation reduction, xenobiotic metabolism, to anatomic structure development, while underexpressed genes were enriched for biological pathways involved in the inflammatory and immune response.

#### DEGs in the sputum of cigarette smokers vs. E-cigarette users

Comparison of CS to e-cigarette users revealed 468 differentially expressed genes in sputum (Fig. 4E and Supplemental Table 4). One hundred and forty-three genes overlapped with genes differentially expressed in the sputum of CS compared with controls (Fig. 4D). Overexpressed genes were overrepresented in biological processes involved mostly in cellular response to stimulus, cell proliferation, tube morphogenesis and oxidative stress, while underexpressed genes were overrepresented in biological pathways associated with the inflammatory and immune response similarly to the comparison between CS and controls.

We compared our findings with published proteomics studies of the sputum of tobacco users [32], [35], [36] (See Supplemental Fig. 2 and Supplemental Table 6). We found the overlap of 23 genes/proteins with those studies. Some of these genes, such as those involved in neutrophil degranulation and antimicrobial humoral response (C3, CLU, B2M, LIZ, CSF1R, and S100A8), response to external stimuli (G6PD, NQO1, and ALDH3A1), and endocytosis (CLU, C3, and B2M), are involved in biological processes affected by exposure to tobacco. We also performed qRT-PCR of selected genes from sputum to validate our findings; results are presented in Supplemental Fig. 3.

### WGCNA analysis and IPA

To determine whether specific gene networks were associated with phenotypic features and tobacco product exposure we performed Weighted Gene Correlation Network Analysis (WGCNA) of the blood and sputum. Gene modules that demonstrated association with a relevant trait then were analyzed using IPA.

#### Blood WGCNA and IPA

**Fig. 5:**
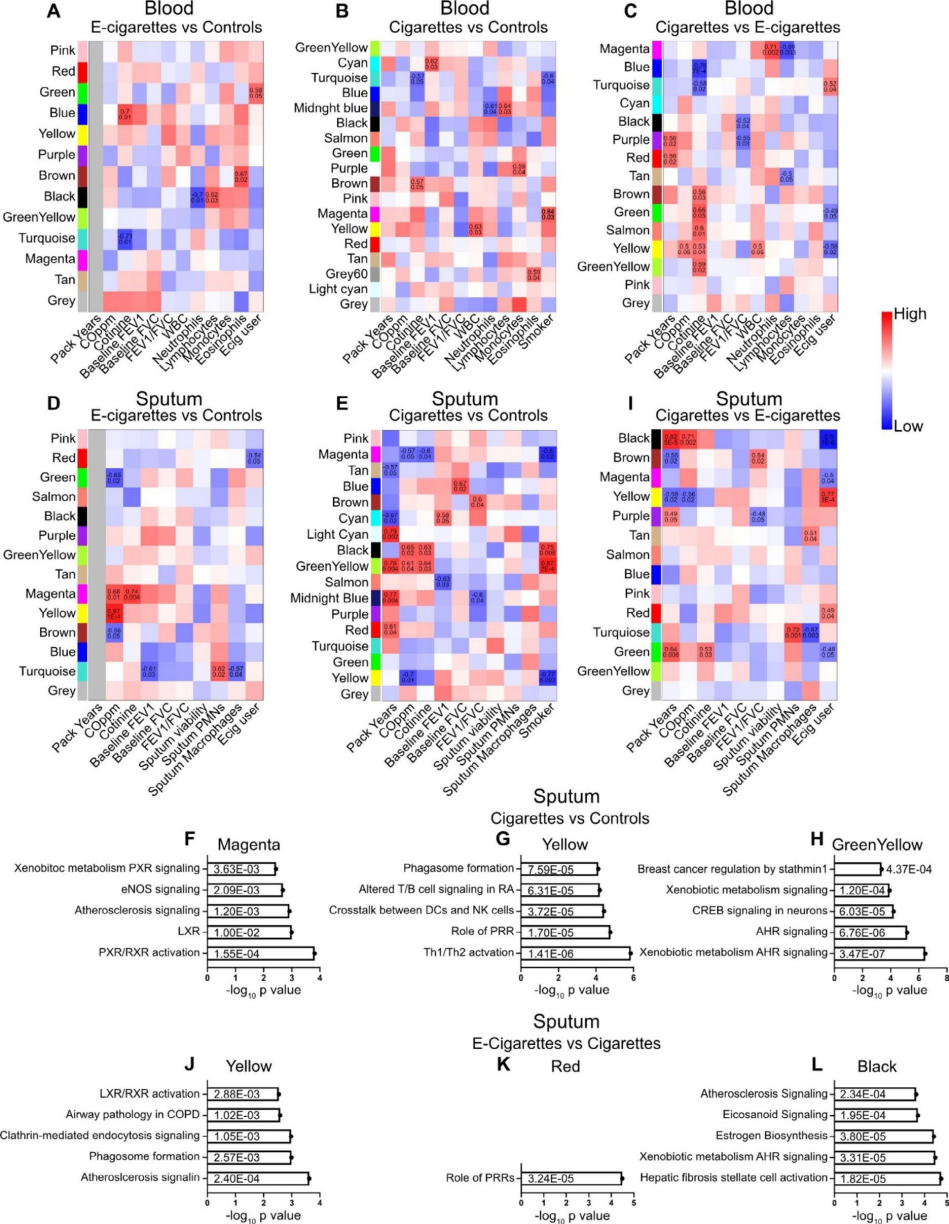
Weighted gene co-expression network analysis identifies modules and canonical pathways associated with type of tobacco product and clinical traits. (A-E and I) Heatmaps showing WGCNA gene modules associated with phenotypic characteristics, including the type of tobacco product exposure (last column in each heatmap) for pairwise comparison in the blood and sputum. (F-G and J-L) Bar plots highlighting IPA canonical pathways from the gene modules associated with the tobacco product type in sputum for (F-H) cigarettes versus controls and (J-L) e-cigarettes versus cigarettes

Multiple gene modules were associated with clinical traits including pack year history, cotinine concentration, and multiple blood cell population counts (Supplemental Tables 9 and Fig. 5A-C). Serum cotinine was associated with the Turquoise module in both e-cigarette users (R = 0.7, p = 0.01) and CS (R = 0.6, p = 0.05), suggesting that in the blood these genes are affected by nicotine exposure. Two modules were significantly associated to CS when compared to controls (Magenta and Turquoise [R = 0.64 and 0.60, p = 0.03 and 0.04 respectively]) (Fig. 5B). The genes in these modules were enriched in biological pathways associated with xenobiotic metabolism, oxidative stress, and pulmonary healing (Supplemental Table 7).

One module (Green module R = 0.58, p = 0.05) was associated with e-cigarettes when compared to controls (Fig. 5A) but no significant biological pathways were identified through IPA. (Supplemental Table 7). However, when we compared CS to e-cigarette users, 3 modules were significantly associated with e-cigarette use (Turquoise, Green and Yellow [R = 0.52, -0.49, -0.67 and p = 0.04, 0.05 and 0.02 respectively]) with genes that enriched in several biological pathways including ferroptosis, eNOs, macropinocytsis and EGF signaling, suggesting that those pathways were differently affected by type of tobacco product.

#### Sputum WGCNA and IPA

Like our analysis of blood samples, multiple gene modules were associated with serum cotinine and eCO suggesting that these gene modules are highly involved in pathways associated to tobacco use (see Fig. 5, Supplemental Tables 8 and 9). When compared to controls, cigarette smoking was associated with 4 gene modules (Magenta, Black, Green-Yellow and Yellow [R = -0.8, 0.75, 0.87 and − 0.77, p = < 0.01, < 0.01, < 0.01 and < 0.01 respectively]). The genes in these modules were overrepresented in biological pathways related to xenobiotic metabolism, oxidative stress response, aryl hydrocarbon receptor (AHR) signaling, coronavirus replication and Th1/Th2 activation (Supplemental Table 8). The comparison between e-cigarettes and controls, showed that one gene module was associated with e-cigarette use (Red [R = -0.54, p-value = 0.05]), with genes involved in the SNARE and synaptogenesis signaling pathways which regulates vesicles fusion and exocytosis, matching other in vitro reports from bronchial epithelial cells [37]. By contrast the comparison between CS and e-cigarettes users in the sputum showed 5 gene modules correlated with type of product use (Black, Magenta, Yellow, Red and Green [R = -0.9, -0.5, 0.77 and 0.49 with p-value = < 0.01, < 0.01, < 0.01, < 0.01 and 0.01 respectively]). Genes from these modules are overrepresented in multiple biological pathways including hepatic fibrosis, xenobiotic response, nicotine degradation, inhibition of metalloproteases, atherosclerosis, and COPD (Supplemental Tables 8 and 9). Suggesting that the type of tobacco product has different effects on these biological processes and how they may lead to disease.

## Discussion

The purpose of our study was to characterize transcriptomic changes in the sputum and blood of cigarette smokers compared to e-cigarette users and controls. We discovered that cigarette smoking is the main driver of transcriptomic differences between the three groups, with a much stronger impact in the airways than blood. Gene modules associated with type of tobacco product exposure were highly associated with levels of eCO and/or with cotinine concentration, both of which were highest in smokers. These findings support the overarching conclusion that cigarette smoking is likely to perturb more biological processes than e-cigarettes, however, this does not imply that e-cigarettes are harmless or safer than conventional cigarettes.

In fact, several genes were differentially expressed in blood and sputum of e-cigarette users compared to controls. These transcriptomic changes support the hypothesis that chronic use of e-cigarettes can lead to biologically significant changes and, potentially contribute to pulmonary diseases. For example, one of the two genes differentially expressed in the sputum of e-cigarette users, *TBC1D3F*, has been implicated in the differentiation of macrophages and vesicle function and trafficking [38], [39] suggesting that vaping may lead to dysfunction of these immune cells in the airways. The other gene (*PMEL*) encodes for a protein that is highly expressed by cells associated with pulmonary lymphangioleiomyomatosis, a potentially lethal cystic lung disease [33], [40]. Furthermore, PMEL is a component of the melanosome, a type of lysosome-related organelle that is dysfunctional in patients with Hermansky-Pudlak, an inherited fibrotic pulmonary disease [41]. However, it is unknown as to how changes in the expression of *PMEL* may influence pulmonary disease development secondary to e-cigarette exposure.

Our study also showed that two genes (*SPOCD1* and *THBS1*) were differentially overexpressed in sputum and blood among CS when compared to controls. *SPOCD1* has been implicated in pi-RNA-directed DNA methylation, with at least two prior studies linking it with smoke exposure 42–44, and *THBS1* is an integral component of the extracellular matrix where it regulates cell migration, cytoskeletal organization, cell proliferation and apoptosis, and plays a role in the regulation of inflammation and modulation of reactive oxygen species [45], [46]. Changes in DNA-methylation and expression of *THBS1* have been associated with atherosclerosis and cancer among smokers, while other reports have linked overexpression of *THBS1* with higher lung cancer survival [47], [48]. Taken together, these genes may aid our understanding of how cigarette smoking can lead to systemic disease from its effects in the respiratory system.

Our findings in sputum resemble those reported by others demonstrating that smoking cigarettes induces a xenobiotic and oxidative stress response and activation of immune-related genes [35], [36]. However, in contrast with data from shotgun proteomics of sputum [32], we did not see evidence of an altered innate immune response or dysfunctional neutrophils in the sputum of e-cigarette users. Furthermore, our analytical approach allowed us to detect misclassification bias and, using only transcriptomics, we were able to distinguish exclusive e-cigarette users from likely dual users or subjects exposed to smoke. Two other strengths of our study are that only high-quality specimens were used for RNA sequencing as RNA degradation could significantly impact RNA-seq analysis [49], and we recruited subjects with no known history of significant comorbidities, including atopy or asthma. Our subjects also underwent spirometry that confirmed normal air flow with no evidence of obstructive pulmonary disease. A recent study also demonstrated that exposure to e-cigarettes affects the immune homeostasis of the respiratory airways and that this is altered by the generation of e-cigarette [31]. A significant difference with that report is our exclusion of sputum samples with greater than 10% squamous cells. Furthermore, the majority of our study participants (66%) were users of fourth generation e-cigarette devices which reflects the current trends of e-cigarette use in the US [50], [51].

Our findings in the blood are similar to those reported by Tommasi et al., in that cigarette smoking induced more transcriptomic changes in blood leukocytes than vaping, although we did not find a dysregulation of mitochondria-specific genes as reported in their study perhaps due to differences in the cell composition studied (PBMCs vs. whole blood) [52]. Our study shows that cigarette smoking generates a greater transcriptomic response in the respiratory system and in blood than e-cigarettes. However, further studies are necessary to better characterize the long-term clinical significance of these gene expression differences to properly support the use of e-cigarettes as a viable harm reduction strategy for cigarette smokers.

Our study does have limitations, including the relatively small sample size. This was associated with the COVID-19 pandemic hindering recruitment of subjects for a study in which nebulized saline was used for sputum induction and concerns of aerosolization of viral particles. This limitation affects our ability to perform subgroup analysis based on e-cigarette brands, type of device (or generation), nicotine concentration or flavors. Furthermore, we did not collect the time of last use of the tobacco product, but we assume that our results reflect a steady state in the airways and blood as one would expect in chronic tobacco use as subjects reported daily use of their tobacco product for at least one year. In addition, since we are analyzing bulk RNA-Seq on samples with a complex cellular composition, lowly expressed transcripts or highly expressed transcripts in low prevalence cells may have been missed. We did not collect data on secondhand smoke exposure either, which could potentially affect our findings. Indeed, one healthy control had elevated cotinine serum levels (but a normal eCO level), although this is not necessarily proof of tobacco exposure [53], [54]. Nonetheless, our results are valuable as they provide early evidence of specific transcripts in the airways and the blood associated with e-cigarette use in contrast to cigarette smoking.

In summary, cigarette smoking and e-cigarette vaping led to significant transcriptomic changes in the airways and the blood of their users. This transcriptomic impact is higher among CS than e-cigarette users. Some of the transcriptomic changes among e-cigarette users are associated with vesicle trafficking and macropynocitosis, biological functions that are fundamental for proper macrophage function and airway immune response. More research is urgently needed to better characterize the long-term effects of these transcriptomic changes in relationship to tobacco product use.

## Electronic supplementary material

Below is the link to the electronic supplementary material.

Supplementary Table 1

Supplementary Table 2

Supplementary Table 3

Supplementary Table 4

Supplementary Table 5

Supplementary Table 6

Supplementary Table 7

Supplementary Table 8

Supplementary Table 9

**Supplemental Fig. 1.** Overlap of differentially expressed genes in blood and sputum of smokers. Venn diagram of differentially expressed genes in blood and sputum of cigarette smokers when compared to controls. [https://doi.org/10.6084/m9.figshare.19878268 under embargo, private link: https://figshare.com/s/778ecc2fce40553bfdb1]. **Supplemental Fig. 2.** Overlap with published proteomics data from sputum of healthy users exposed to different tobacco products. We compared DEGs from all comparisons (p < 0.05) from our data (RNA) with published significantly different proteins from the sputum of healthy e-cigarette (E-Cigs) or healthy cigarette (Cig) users. A total of 23 genes/proteins overlapped with other publications, 12 with reports from E-cigarette users and 19 from smokers. **Supplemental Figure 3.** qRT-PCR of selected genes in sputum. Relative levels of each gene were normalized against four control genes (*HPRT*, *18s*, *ACTIN* and *GAPDH*). ANOVA was use to compare all three groups and Dunnett’s test was used to test pair comparisons. *=p < 0.05, **=p < 0.01, ***=p < 0.001. A. Data were log10 transformed and plotted as a heatmap. B. Presented as the relative fold change when compared to the four control genes. **Supplemental Fig. 4.** Weighted gene co-expression network analysis gene modules associated with tobacco product type. (A-E) heatmaps of genes present in sputum WGCNA modules significantly associated with tobacco product type (e-cigarettes versus cigarettes). [https://doi.org/10.6084/m9.figshare.19878268 under embargo, private link: https://figshare.com/s/778ecc2fce40553bfdb1]

## Data Availability

The database generated for this study is available by request. For inquiries, please email corresponding author.
