# Supplementary figures and images for: Transcriptomic responses in the blood and sputum of cigarette smokers compared to e-cigarette vapers
Source: Respir Res. 2023 May 18;24:134. doi: 10.1186/s12931-023-02438-x (PMC10196320; doi:10.1186/s12931-023-02438-x)

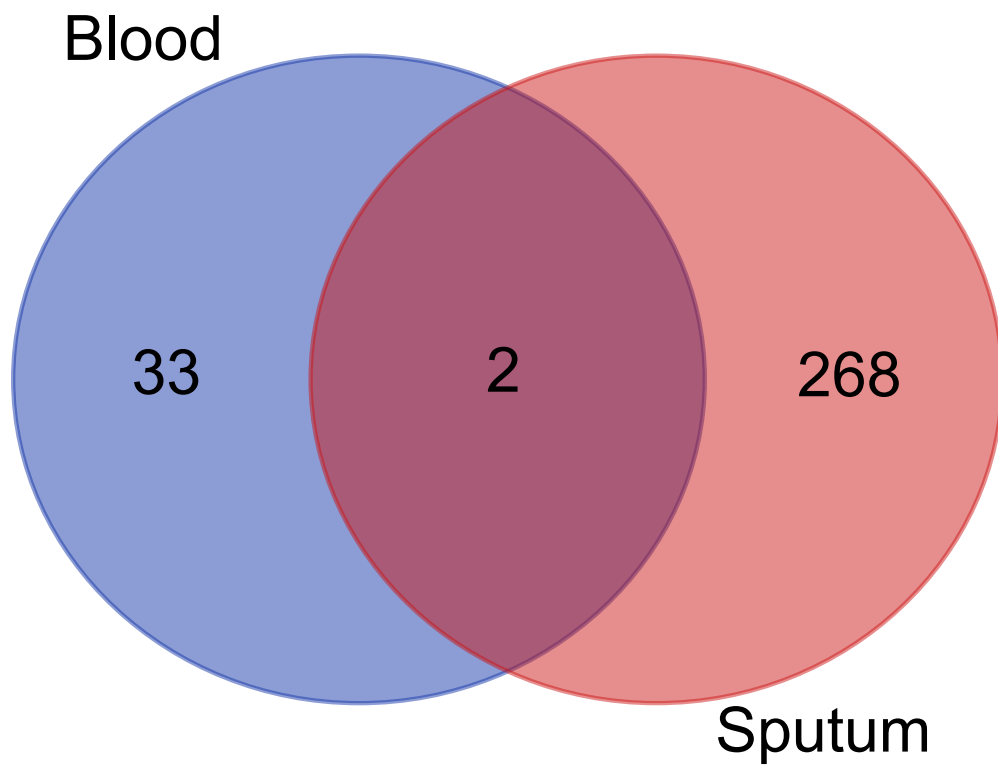

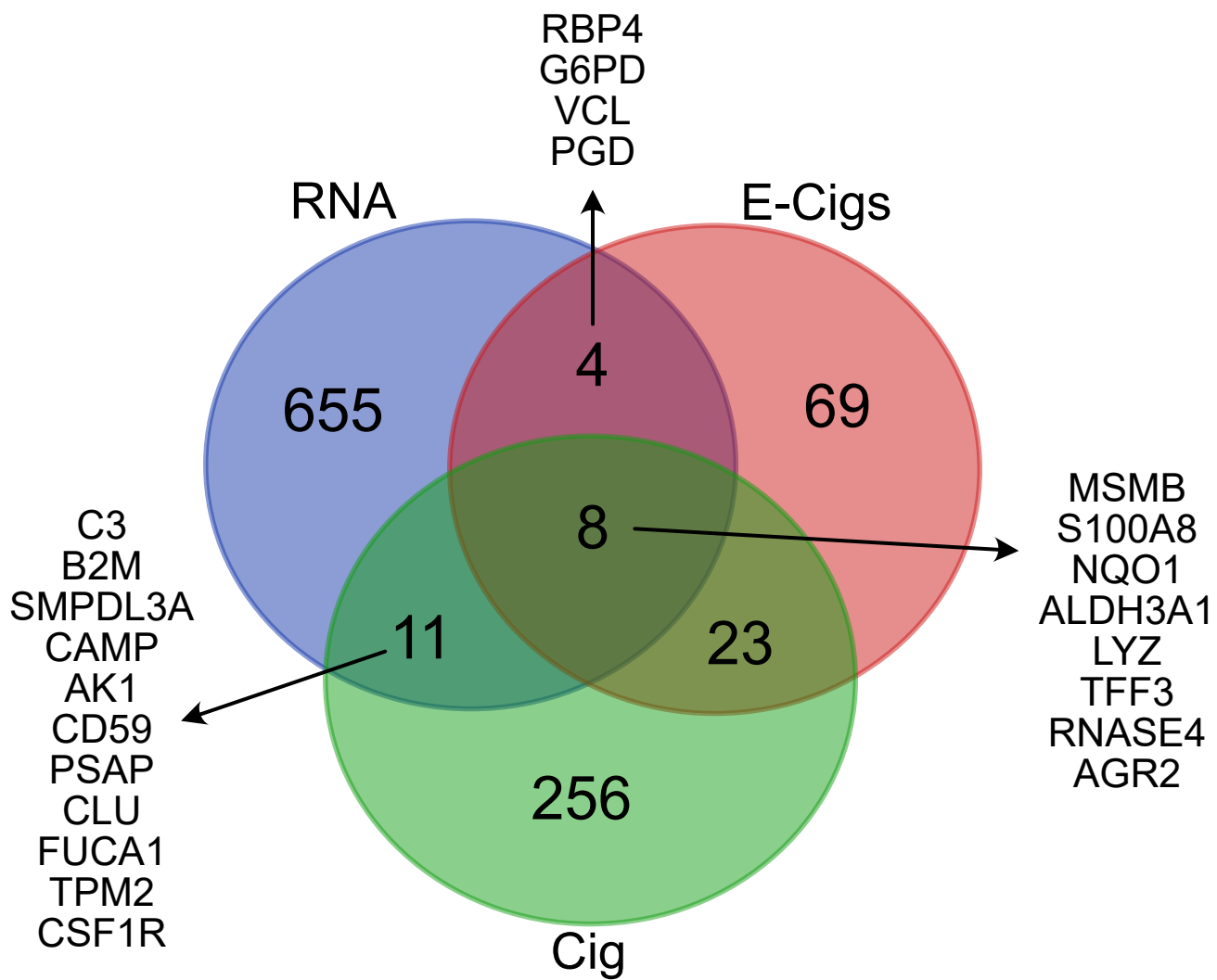

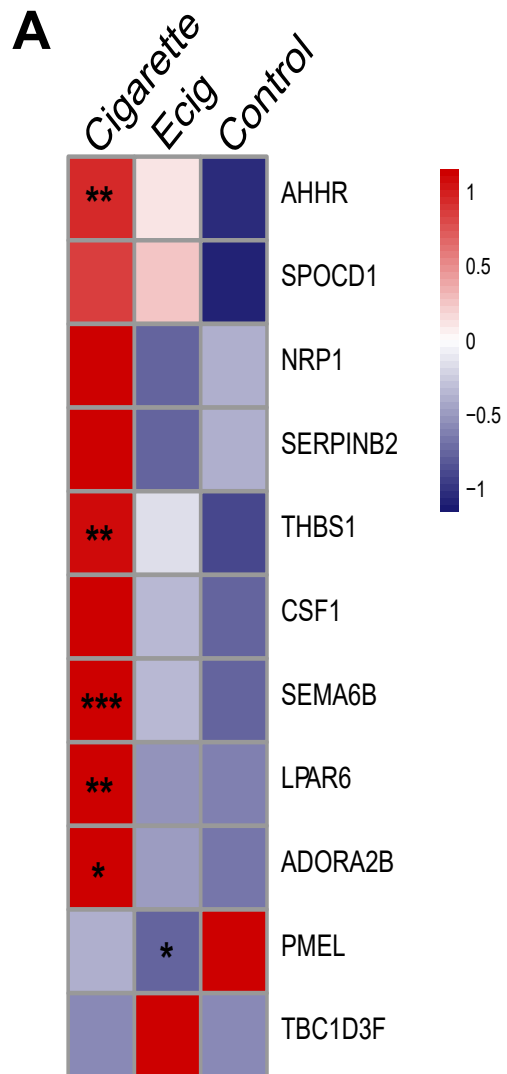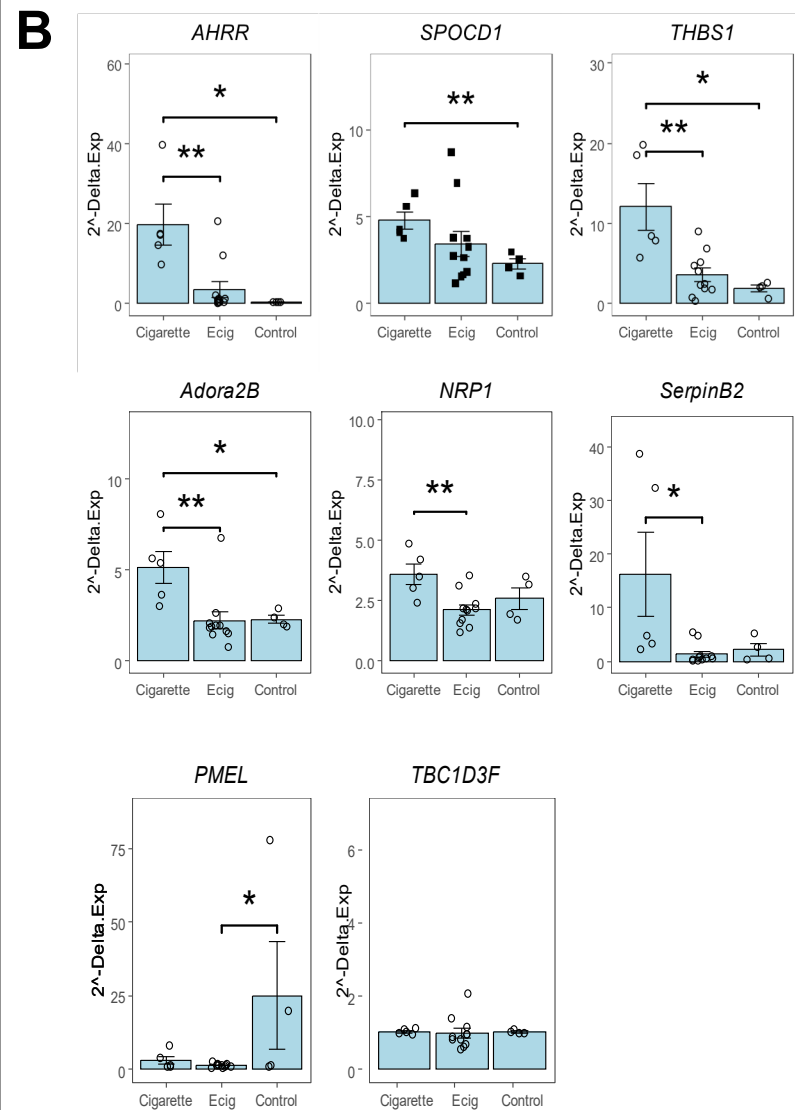

Supplemental Figure 3

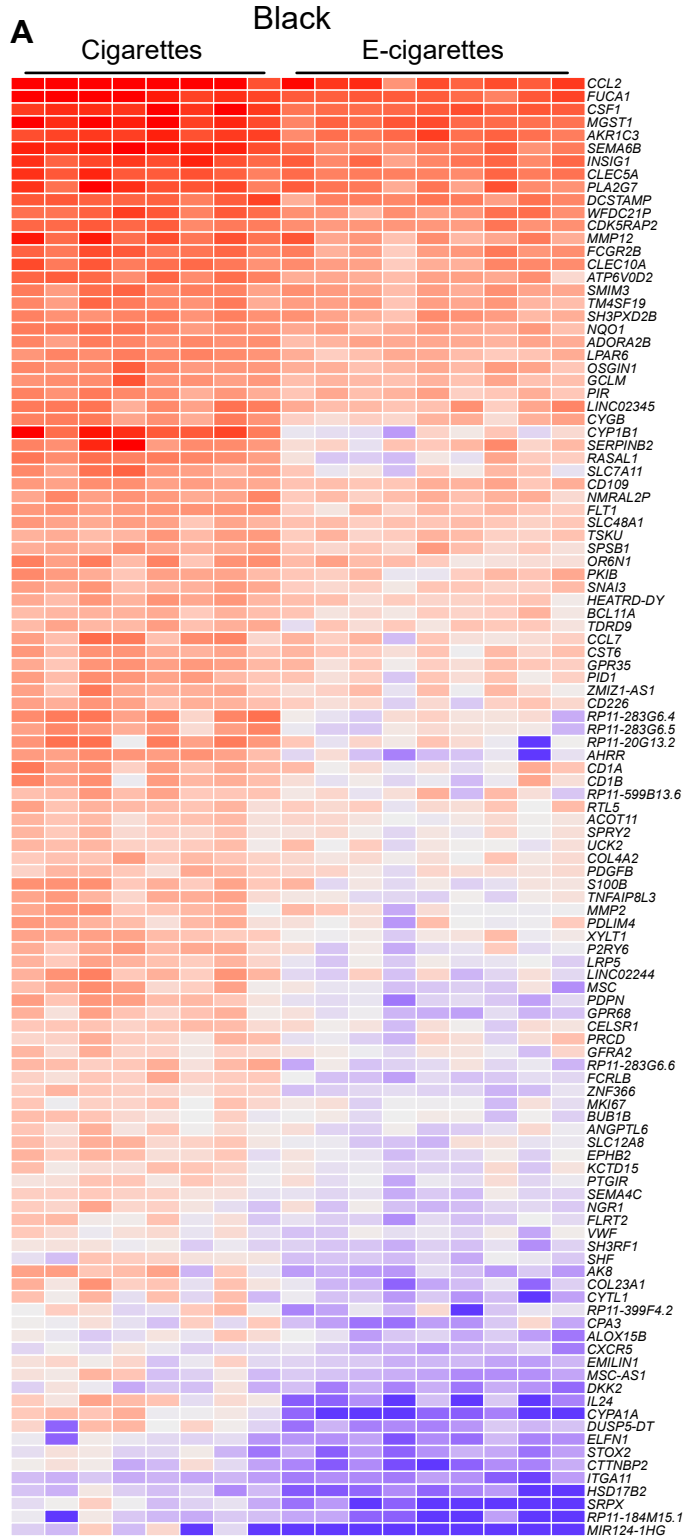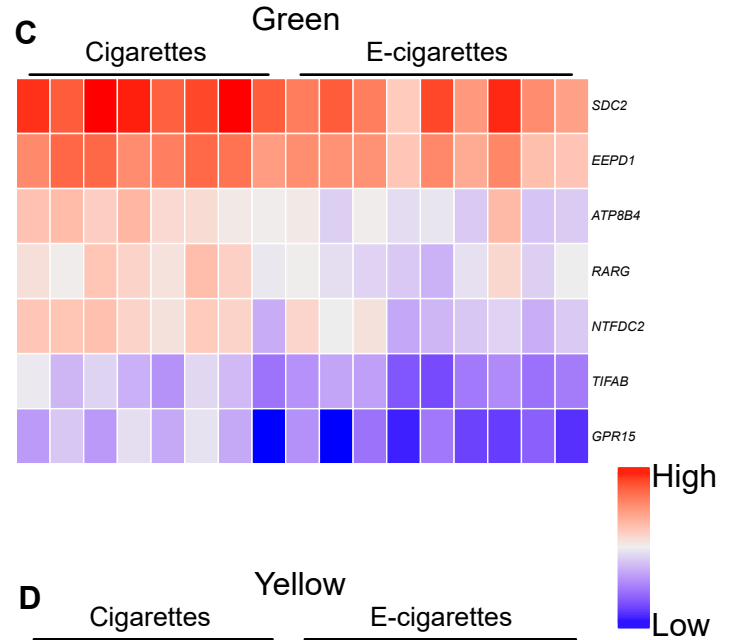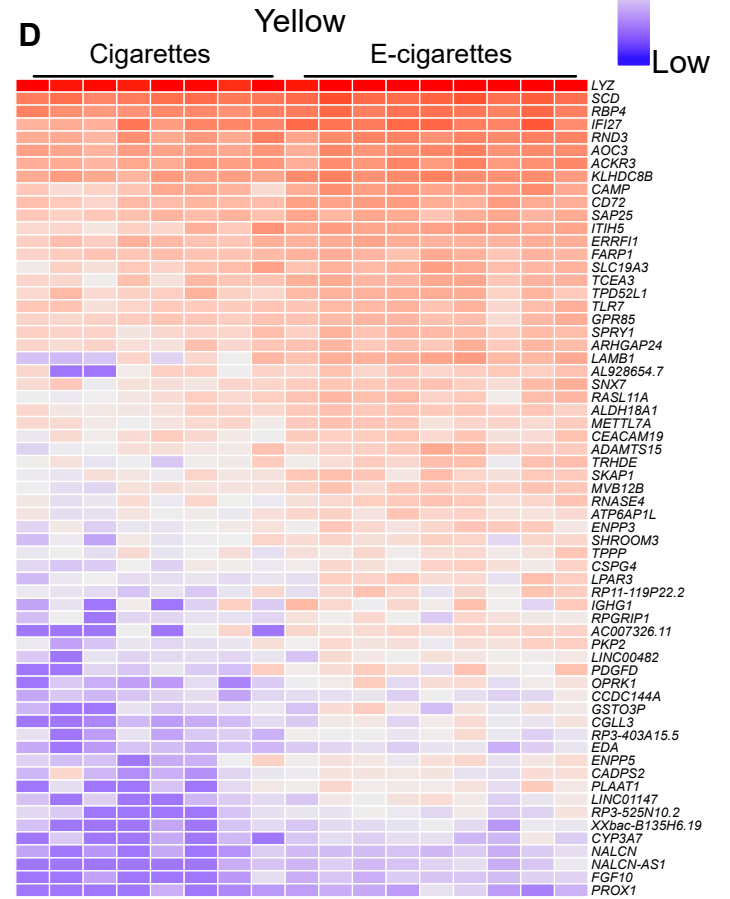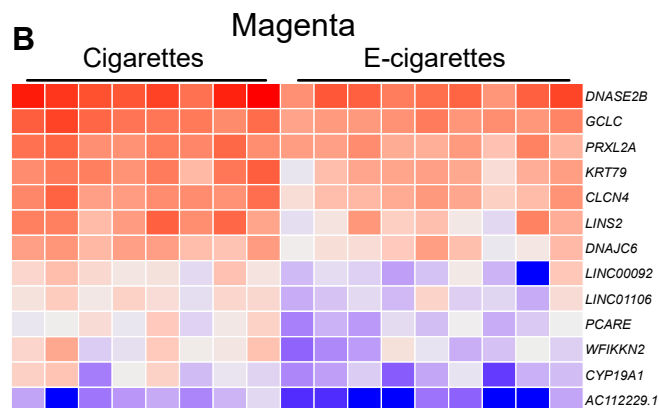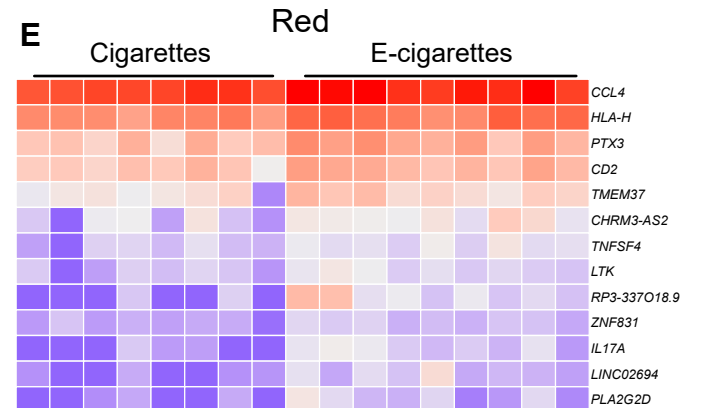

Supplemental Figure 4

Supplement: Supplementary file 10 — Supplemental Fig. 1. Overlap of differentially expressed genes in blood and sputum of smokers. Venn diagram of differentially expressed genes in blood and sputum of cigarette smokers when compared to controls. [https://doi.org/10.6084/m9.figshare.19878268 under embargo, private link: https://figshare.com/s/778ecc2fce40553bfdb1]. Supplemental Fig. 2. Overlap with published proteomics data from sputum of healthy users exposed to different tobacco products. We compared DEGs from all comparisons (p < 0.05) from our data (RNA) with published significantly different proteins from the sputum of healthy e-cigarette (E-Cigs) or healthy cigarette (Cig) users. A total of 23 genes/proteins overlapped with other publications, 12 with reports from E-cigarette users and 19 from smokers. Supplemental Figure 3. qRT-PCR of selected genes in sputum. Relative levels of each gene were normalized against four control genes (HPRT, 18s, ACTIN and GAPDH). ANOVA was use to compare all three groups and Dunnett’s test was used to test pair comparisons. *=p < 0.05, **=p < 0.01, ***=p < 0.001. A. Data were log10 transformed and plotted as a heatmap. B. Presented as the relative fold change when compared to the four control genes. Supplemental Fig. 4. Weighted gene co-expression network analysis gene modules associated with tobacco product type. (A-E) heatmaps of genes present in sputum WGCNA modules significantly associated with tobacco product type (e-cigarettes versus cigarettes). [https://doi.org/10.6084/m9.figshare.19878268 under embargo, private link: https://figshare.com/s/778ecc2fce40553bfdb1] [file 12931_2023_2438_MOESM10_ESM.pdf]
